# Strengthening immunization service delivery post Ebola virus disease (EVD) outbreak in Liberia 2015-2017

**DOI:** 10.11604/pamj.supp.2019.33.2.17116

**Published:** 2019-05-28

**Authors:** Adolphus Clarke, Nicholas Blidi, Joseph Yokie, Mary Momolu, Chukwuemeka Agbo, Roland Tuopileyi, Julius Monday Rude, Mohammed Seid, Yohannes Dereje, Zakari Wambai, Alex Gasasira, Laura Skrip, Ngozi Kennedy, Evans Lablah, Joseph Chukwudi Okeibunor, Mamoudou Harouna Djingarey, Ambrose Talisuna, Ali Ahmed Yahaya, Soatiana Rajatonirina, Ibrahima Socé Fall

**Affiliations:** 1 Ministry of Health, Monrovia, Liberia; 2 World Health Organization Country Office, Monrovia, Liberia; 3 National Public Health Institute, Monrovia, Liberia; 4 UNICEF Country Office, Monrovia, Liberia; 5 World Health Organization, Regional Office for Africa, Brazzaville, Congo

**Keywords:** Immunization, surveillance, Ebola, recovery, vaccines, polio

## Abstract

**Introduction:**

The Ebola virus disease (EVD) outbreak in Liberia from 2014-2015 setback the already fragile health system which was recovering from the effects of civil unrest. This led to significant decline in immunization coverage and key polio free certification indicators. The Liberia investment plan was developed to restore immunization service delivery and overall health system.

**Methods:**

We conducted a desk review to summarize performance of immunization coverage, polio eradication, measles control, new vaccines and technologies. Data sources include program reports, scientific and grey literature, District Health Information System (DHIS2), Integrated Diseases Surveillance and Response (IDSR) database, auto visual AFP detection and reporting (AVADAR) and ONA Servers. Data analysis was done using Microsoft excel spreadsheets, ONA software and Arc GIS.

**Results:**

There was a 36% increase in national coverage for Penta 3 in 2017 compared to 2014 from WUENIC data. Penta 3 dropout rate reduced by 2.5 fold from 15.3% in 2016 to 6.4% in 2017; while MCV1 coverage improved by 23% from 64% in 2015 to 87% in 2017. There was a rebound of non-polio AFP rate (NPAFP) rate from 1.2 in 2015 to 4.3 in 2017. Furthermore, there was a 2-fold increase in the number of AFP cases receiving 3 or more doses of OPV from 36% in 2015 to 61% in 2017.

**Conclusion:**

Liberia demonstrated strong rebound of immunization services following the largest and most devastating EVD outbreak in West Africa in 2014 - 2015. Immunization coverage improved and dropout rates reduced. However, there are still opportunities for improvement in the immunization program both at national and sub-national levels.

## Introduction

When implemented under optimal conditions, immunization is one of the most successful and cost-effective health interventions against vaccine preventable diseases [1] saving an estimated 2-3 million lives annually around the world [1, 2]. Such success has been largely attributed to the launching of the expanded program on immunization (EPI) by the World Health Organization in 1974 with the aim to reduce morbidity and mortality associated with six major causes of death among children [1, 2]. Immunized children can lead to healthier and more productive lives [1, 3] and extending vaccination schemes to adolescents and adults can propagate these advantages through the life course [3, 4]. The global vaccine action plan 2011-2020 (GVAP) was designed with a vision for the decade of vaccines (DoV), to eradicate, eliminate or control serious, life-threatening or debilitating vaccine preventable diseases [4, 5]. Liberia has been committed to implementing universal immunization coverage through the GVAP to achieve the goal of a DoV. Despite a decade-long civil war that decimated health infrastructure, institutions and overall economy, the Ministry of Health (MOH) made steady efforts to revitalize the health system. The immunization program was strengthened with marked increases in coverage and immunity [6-8]. The 2014-2015 outbreak of Ebola virus disease (EVD) set back an already fragile health system that was recovering from the civil unrest. During the EVD outbreak, less than of 70% of health facilities were open and universally demand for health services was low due to fear and distrust in the health system. The Government of Liberia (GOL) declared a state of emergency to control the wide spreading EVD outbreak [9]. Subsequently, all planned routine immunization activities such as outreach, supplemental immunization activities (SIAs), the Human Papilloma Vaccine (HPV) demonstration project, and polio vaccination campaigns, among others, were paused [6-8]. These were in accordance to the guidance for immunization programs in the african region in the context of Ebola [10]. On the same note, the MOH could not implement and monitor the annual EPI work-plans, as all attention, including deployment of human resources were refocused to control the outbreak. Similarly, VPDs surveillance including specimen transportation by DHL was neglected at the height of the EVD outbreak. Thus, Penta-3 immunization coverage decreased by 26%, from 76% in 2013 to 50% in 2014 while, measles containing vaccine (MCV) coverage declined from 74% in 2013 to 58% in 2014 [11]. Simultaneously, there was a significant drop in the Non-polio AFP rate (population under 15 years) from 2.9/ 100,000 in 2013 to 1.2 /100,000 in 2015 (IDSR Database), well below polio free certification levels. Guinea and Sierra Leone encountered similar decline in immunization service delivery [12, 13]. Due to disruptions of health service provision, approximately 20, 000 children were unvaccinated on a monthly basis during the EVD outbreak, contributing to about 1.5 million unvaccinated children over an 18-month period [12]. Post EVD, the Liberia investment plan for rebuilding resilient health systems 2015-2021 and the immunization recovery plan for Liberia were developed with the aim to restore the immunization service delivery and overall health system. The introduction and piloting of new vaccines, technologies and innovations to strengthen quality health service delivery were also integral to the investment plan [6-8]. This paper aims to describe Liberia immunization program performance following EVD outbreak in 2014-2015, summarizing program management, activities and new vaccines & technologies implemented to strengthen EPI program, highlighting challenges and suggest recommendations for improvement plans to consolidate on progress made.

## Methods

We conducted a desk review to summarize key activities conducted to restore immunization service delivery based on the strategic goals of the GVAP 2011-2020 namely: vaccine coverage targets at country and district levels, polio eradication, measles and MNT elimination, introduction of new vaccines and technologies (Table 1).

**Table 1:** Indicator description and target of the Polio eradication used in Liberia

Indicators	Description	Target	Comments
**Penta 3 Coverage**	Percentage of target children (< 1 year) receiving Penta 3 vaccine per year for routine immunization	80%	The national target was reset to 75% in 2015 following EVD outbreak. However, it will steadily increase to GVAP target of 95% by 2020
**Penta 3 Dropout rate**	Difference between Penta 1 and Penta 3 expressed as a rate	<=10%	
**Polio coverage**	An objective measure of SIA quality that can be used to guide improvements to reach more children by enabling corrective action both during SIAs using independent monitors	95%	
**Polio Lots Quality Assurance Sampling (LQAS) Survey**	A random sampling methodology as a method of quality control	< 3 out 60 missed children per Lot	
**Measles SIA coverage**	Percentage of target children (<5years) receiving Measles vaccine during SIA	95%	
**Non-Polio AFP rate**	Measures the sensitivity of AFP surveillance system	2/100, 000 pop. < 15 years	
**AFP stool Adequacy**	Measures the completeness of case investigation and quality of AFP stool specimen for laboratory testing	80%	

**Study setting:** Liberia is a tropical country in West Africa with an estimated population of about 4.1 million people, annual growth rate of 2.1%, total land area of 111,370 km2, and is bordered by Sierra Leone in the west, Cote d’Ivoire in the East, Guinea in the North and the Atlantic Ocean in the South. There are 15 counties (equivalent to WHO districts) and 91 health districts (sub-districts). It has 570 health facilities delivering EPI services across the 15 counties. The EPI manager leads the program with team leads for immunization, VPD surveillance, SIA, Logistics and cold chain. Child Survival Focal Person (CSFPs) at county and district levels manage the program at the levels and supervises the vaccinators at the health facilities.

**Data sources:** data sources included immunization program evaluation and joint appraisal reports, program audits, technical reports, weekly and monthly EPI and VPD surveillance bulletins, quarterly EPI review meeting reports, meeting minutes, scientific literature, and literature. We also sourced data from DHIS2 database, IDSR database, auto visual AFP detection and reporting (AVADAR) and ONA Servers. Surveillance data were collected using the open data kit (ODK) mobile data application.

**Data analysis:** data on programs and vaccine uptake were evaluated for the period of 2015-2017 to show the impact of post-EVD implementation of the Liberia investment plan and immunization recovery plan. Frequencies and percentages were calculated to describe trends in coverage over time and geographic location. Data analysis for the ISS checklist was automatically performed in the ONA servers or exported to Microsoft Excel for further analysis. ArcGIS was used to develop geospatial and temporal distribution of data, including proximity analysis. We triangulated administrative data, independent monitoring data, LQAS survey data and WUENIC to compare outcomes and inform further analysis.

## Results

### Routine immunization

The development of immunization recovery plan post EVD in 2015, the revision and implementation of routine immunization micro plans based on principles of RED/REC and the implementation of urban immunization strategy in Montserrado County to reach urban slums and underserved population were the main activities to increase the access to the immunization services. As part of capacity building of the vaccinators, Immunization in Practice (IIP) training was conducted for health workers, refresher trainings, on-the-job training and mentorship for vaccination teams. The improvement of supply chain was done though the completion of the national vaccine store at Caldwell, Montserrado and two regional cold rooms in Grand Gedeh and Bong Counties. The optimization of the cold chain equipment and recruitment and training of 15 county supply chain officers were also part of the strategy. As part of the management of the program, besides development of the comprehensive multi year plan (cYMP) 2016-2020, the budget allocation was increased from $50, 000 USD in 2015 to $650, 000 USD in 2016. Capacity building for national teams was done through trainings and workshops. The country introduced electronic supervision tools such as electronic integrated supportive Supervision (ISS) and electronic surveillance (eSurv) checklists via open data kit (ODK) and WHO AFRO ONA servers and enhanced community involvement through advocacy meetings, social mobilization, deepened community engagement. In addition, technical Working Group Meetings, supportive Supervisions and quarterly EPI review meetings were conducted besides the development and dissemination of information products such Monthly EPI bulletins, VPD surveillance updates.

### Polio eradication

The program working with GPEI partners developed the Brazzaville Initiative (B.I.) to re-invigorate polio eradication activities following EVD outbreak control. The B.I. followed a mid-term review of the GPEI strategic plan in mid-2015 recommending strengthening AFP surveillance and containment activities, improving the quality of immunization campaigns and building national capacity to respond to outbreaks. The national polio committee prepared and presented the annual national polio update for 2016 following the EVD outbreak control at the ARCC annual certification meeting in Malabo, Equatorial Guinea in 2017, which was accepted. Active case search for AFP and stool transport for laboratory confirmation in Abidjan via DH was re-established In addition, OPV SWICTH was conducted in 2016 and IPV introduced into routine immunization in 2017. GAP 1 a and b were completed in 2016.

### Disease control programs

The country conducted a catch up Measles SIAs targeting children 6-59 months in May 2015 and strengthened measles surveillance within the integrated disease surveillance response (IDSR) strategy. The analysis of low yellow fever EPI coverage in 3 counties: Grand Bassa, Rivercess, and Montserrado were conducted and improvement plans developed.

### New vaccines and technologies

The country introduced new vaccines such Rota vaccine, IPV and HPV Demo Project. Mobile data collection tools and GIS technologies, namely open data kit (ODK), ONA and AVADAR GIS software was also introduced.

### Outcome results

**Figure 1:**
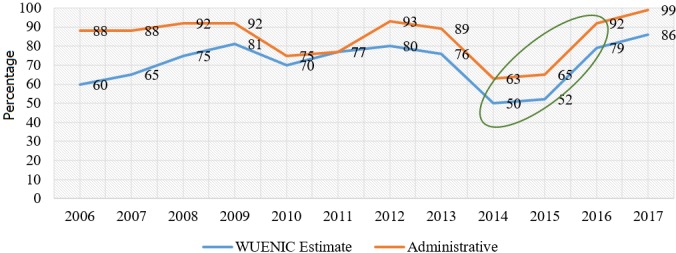
Trends of Penta 3 coverage from 2006 to 2017, Liberia

**Routine immunization of Penta 3:** there was a 34% increase in immunization coverage for Penta 3 from 52% in 2015 to 86% in 2017 (WUENIC 2017) (Figure 1). The trends for both administrative coverage and WUENIC follow similar pattern but there are discrepancies between coverage rates reported by both administrative and WUENIC reports. However, the absolute number of children 0-11 months vaccinated with Penta 3 in 2017 was 165, 972, an 11% increase in number of vaccinated children from 150, 231 in 2016 (DHIS2 database). Table 2 shows that at the national level, Penta 3 dropout rate in Liberia reduced approximately 3 fold from 15.3% in 2015 to 5.5% in 2017. In 2015, 80% of Counties had dropout rates above 10% compared to 20% of counties by 2017. In 2017, Grand Gedeh and River Gee Counties had negative dropout rates. However, Grand Kru, Lofa, Margibi Counties had dropout rates close to 10%, although this was lower than it had been for Grand Kru and Margibi in 2015. Grand Bassa and Bomi counties consistently had dropout rates above 10% for the period under review. Further analysis shows that Grand Cape Mount, Grand Gedeh, Grand Kru, Margibi, Montserrado, Nimba, River Gee, Rivercess and Sinoe counties had two-fold reduction in dropout rates from 2015 to 2017.

**Table 2:** Penta 3 dropout rate (DOR) at national and sub-national levels from 2015 to 2017, Liberia

County	Penta 3 DOR 2015	Penta 3 DOR 2016	Penta 3 DOR 2017
Bomi	16.50%	26.10%	10.40%
Bong	9.10%	7.00%	4.60%
Gbarpolu	10.70%	6.90%	6.10%
Grand Bassa	22.70%	22.00%	15.10%
Grand Cape Mount	19.90%	15.40%	9.20%
Grand Gedeh	8.10%	1.10%	-1.10%
Grand Kru	22.80%	19.50%	8.00%
Lofa	6.20%	1.50%	9.10%
Margibi	24.80%	11.40%	10.60%
Maryland	13.80%	7.60%	7.30%
Montserrado	14.30%	11.20%	4.00%
Nimba	20.50%	8.50%	1.30%
River Gee	19.80%	8.70%	-9.00%
Rivercess	14.30%	11.60%	7.10%
Sinoe	10.00%	7.30%	2.30%
Liberia	15.30%	10.70%	5.50%

**Polio eradication:** there was a significant improvement in AFP surveillance indicators with a rebound of non-polio AFP rate (NPAFP) rate from 1.2 in 2015 to 3.7 in 2016. As of Epi Week 52, 2017, the NPAFP rate was 4.3. The stool adequacy rate also improved from 79% in 2016 to 82% as of Epi Week 52, 2017, meeting the global target of 80%. Furthermore, there was 2 fold increase in the number of OPV doses received among the AFP cases from 36% in 2015 to 61% in 2017. However, 20% (32/168) of the cases reported unknown number of OPV doses received. The LQAS survey results showed that a total of 10/15 (67%) of Lots were accepted in RD3 compared to 9/15 (60%) of Lots RD1 of Polio campaign in 2017. Only 0.1% Lot were rejected at < 80% in RD3 campaign compared to 33% in RD1 campaign 2017. However, Maryland, Margibi and Sinoe counties (Lots) were consistently rejected in the LQAS surveys conducted in 3 round of polio campaigns in 2017 (Table 3). IM assessment for Polio campaigns showed that 3/3 (100%) of the campaigns in 2017 and 2016, had estimated coverage above 95% (less than 5% of target children missed) compared 0% of the same indicator in 2015. In 2016, IM results of all four rounds in 2016 and 2017 showed that less than 5% of children were missed during the polio campaigns, converging with administrative data for the national level (Table 4).

**Table 3:** Showing LQAS survey results from RD1- RD4 Polio campaigns, 2017 by County, Liberia

County	LQAS Feb 2017	LQAS Mar 2017	LQAS Nov 2017	LQAS Dec 2017
Bomi	Accepted	Accepted	Rejected	NA
Bong	Accepted	Accepted	Accepted	NA
Gbarpolu	Accepted	Accepted	Accepted	NA
Grand Bassa	Accepted	Accepted	Accepted	NA
Grand Cape Mount	Accepted	Accepted	Accepted	Rejected
Grand Gedeh	Accepted	Rejected	Accepted	Accepted
Grand Kru	Rejected	Accepted	Accepted	NA
Lofa	Rejected	Accepted	Accepted	Rejected
Margibi	Accepted	Rejected	Rejected	Accepted
Maryland	Rejected	Rejected	Rejected	Rejected
Montserrado	Accepted	Rejected	Accepted	Accepted
Nimba	Rejected	Accepted	Accepted	Rejected
Rivercess	Rejected	Accepted	Accepted	Accepted
River-Gee	Accepted	Accepted	Rejected	NA
Sinoe	Rejected	Rejected	Rejected	NA

Data source (WHO Liberia database and WHO AFRO ONA Server)

**Table 4:** Administrative coverage and Independent Monitoring (IM) results for Polio campaigns in 2016 and 2017, Liberia

	2016		2017	
Polio SIA Round	Administrative Coverage	Independent Monitoring	Administrative Coverage	Independent Monitoring
RD 1	96.60%	95.20%	99%	97.4%
RD 2	95.20%	95.80%	99%	97.4%
RD 3	96.00%	96.40%	96.4%	97.7%
RD 4	99.90%	96.60%		

Data source: MOH administrative database and WHO IM database

**Figure 2:**
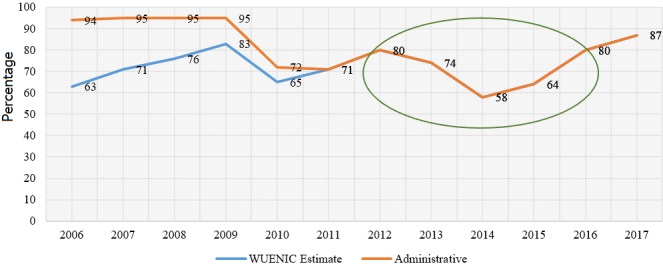
Graph showing trends of MCV1 coverage 2006 to 2017

**Measles control:** Figure 2 shows that MCV1 coverage improved by 23% from 64% in 2015 to 87% in 2017 based on WUENIC 2017 data. Furthermore, Table 5 also shows that in 2015, the post measles campaign coverage survey at the national level was 73.1% by vaccination card and verbal history while the coverage of 90.4% by verbal history alone. Only 3/15 (20%) counties had vaccination coverage above 90%. However, 11/15 (73%) of counties achieved coverage above 90% when evaluated by verbal history alone. The 3 most populated counties Lofa, Nimba, Montserrado, had coverages below 65% when assessed by vaccination card alone. A significant number of suspected measles cases were reported in Liberia in 2017. Of the reported cases, 17% (317/ 1818) were laboratory confirmed, 47% (858/ 1818) tested negative for measles. Of the 814 discarded measles cases, 40% (347/ 858) tested positive for rubella. The pie chart shows that 29% of confirmed measles cases during the review period received MCV1. A significant number (56%) of the reported cases had unknown vaccination status. As of Epi week 52, 2017, highly populated counties (Nimba, Bong and Montserrado) accounted for most measles outbreaks in the country. Nimba and Bong also share common borders with neighbouring countries such as Guinea and Cote d’Ivoire. Montserrado County accounts for 1/3 of the country population, with urban slums and population migration.

**Table 5:** Showing results of measles coverage survey 2015

County	Coverage by vaccination card (%)	Coverage by verbal history alone (%)
Grand Kru	99.4	99.2
Grand Gedeh	95.4	99.3
River Gee	92.6	97.6
Bong	82.8	95.5
Margibi	80.4	94.8
Rivercess	81.1	94.2
Sinoe	76.5	94.4
Grand Bassa	76.7	93.1
Monrovia	77.3	90.7
Maryland	74.1	90.7
Bomi	69	89.9
Nimba	61.9	90.3
Lofa	58.3	87.1
Montserrado	43	79.8
Grand Cape Mount	43.5	72.4
**Total**	**73.1**	**90.4**

**Data source:** Liberia Post Measles Campaign coverage survey 2015

**Figure 3:**
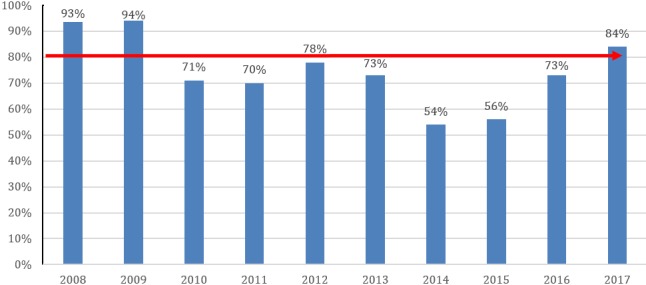
Yellow fever vaccination coverage by year from 2008- 2017, Liberia (Data Source WUENIC 2017 and Admin (2017))

**Yellow fever control:** there was significant improvement in yellow fever vaccine (YF) coverage in 2017 which was 84%. The YF vaccine coverage for routine immunization was below target levels from 2010 up to 2016 (Figure 3). Disaggregated data by county shows that 60% (9/15) of counties met the national coverage target in 2017. Only 47% (7/15) met the national target in 2016 and 2017. Grand Cape Mount, Grand Gedeh, Maryland and River Gee counties consistently had lower coverage from 2015 to 2017.

## Discussion

Our study shows that IDSR has been actively implemented in Liberia since 2015. Feedback to the counties and HCFs, including laboratory results using email, regular supervision to all the HCFs, the introduction of mobile phone applications for data collection and management and the electronic platform for AFP surveillance were some of the innovations observed during this period. The electronic surveillance is useful in reducing the time from detection to reporting of public health events, allowing a fast investigation and response [14]. Although the electronic surveillance was piloted in Liberia, a study conducted in Tanzania in 2012 [15] and Kenya in 2016 [16] demonstrated that the use of mobile phones for surveillance can dramatically improve the timeliness and completeness of the reports. The reestablishment of IDSR in Liberia utilized the existing health structure through integration of existing surveillance systems. This included establishment of a coordination mechanism to link various surveillance systems to create an integrated system, harmonization of data collection tools, procurement of standardized data storage; and storing data in a uniform database where could easily be accessed by users and policy makers. This integrated system is particularly important to address all the public health events notifiable under IHR (2005) [17]. However, Liberia was running a parallel system (HMIS) to report monthly other conditions together with the conditions reportable under IDSR, leading to data discrepancies. The same challenge was observed during an assessment conducted in Ethiopia [18] and was even worse in Tanzania in 1998, when five parallel surveillance systems lead to poor performance in all the IDSR functions [19].

The implementation of IDSR in Liberia guided the decision-making for public health action and contributed to the overall health sector goal of reducing morbidity and mortality due to preventable causes, exemplified by all the outbreaks suspected, confirmed and controlled quickly with law case fatality rate in 2017. However, although malaria was the most important cause of admission and deaths in Liberia, especially among children [12, 20], it was not part of IDSR. In other countries like Ghana [21] and Ethiopia [18], the use of malaria data obtained through IDSR lead to important decisions to reduce the morbidity and mortality of the illness. The resilience of surveillance system in Liberia was also contributed by the integration of the private HCFs into IDSR, considering that 38% (296/773) of the HCFs in Liberia were private, with 96% (285/296) of them reporting regularly to the surveillance system. Unlike Liberia, the integration of private facilities into surveillance system in other countries was a challenge, compromising the completeness of reporting [13]. Despite the temporary interruption in reporting by the DSOs and ZSOs verified in 2017 in Liberia, the completeness of reporting remained high in 97%, since the private facilities continued reporting and ZSOs from Montserrado were supported by other partners. However, it affected negatively the number of supervisions conducted to the HCFs, being 50% (384/773) from September to November, 2017, far from the 80% recommended in IDSR guidelines. The consistent high completeness and timeliness of reporting was also contributed by the availability of resources like computer, printers and means of transport provided by WHO at district and county level. However, the same resources still not available at facility level in Liberia. The lack of resources, including reporting forms verified in Nairobi [22] and Nigeria [23] compromised the submission of weekly reports and other IDSR functions in general.

Although all the HCFs in Liberia had IDSR focal person, the follow up trainings were poor, representing only 46% (178/384) of IDSR focal point trained in the past year due to attrition of the previously trained staff. This may lead to poor quality of reporting, difficulties to use the standard case definition (relying more in clinical judgment), poor data analysis skills and inadequate supervisions and feedback to the community level as demonstrated in Tanzania in 2002 [24]. Liberia also had not integrated yet into IDSR other components of IHR (2005) like animal, food poisoning, chemical poisoning and disaster in the same level as other countries [17]. Instead, those events were classified as cluster of events and deaths. The qualitative questions from IDSR supervision revealed that the health workers were not satisfied with their level of training in IDSR, besides the workload, poor feedback with the issues raised in the supervision not addressed before the following supervision and inadequate means of transport and communication. The same findings were observed in qualitative study conducted in Zambia in 2016 [25]. Our study had several potential limitations. There was no information available about the implementation of IDSR in Liberia before the EVD outbreak. In addition, after the re-introduction of IDSR in 2015, different supervision tools were used, making difficult to compare the progress of indicators over the years. The assessment was also done by people from the county (DSOs) and information biases are expected. The interviews with the OICs were not recorded and transcribed, making impossible to reproduce verbatim quotes in our analysis. Despite these limitations, our findings can reliably be used for decision making and documentation.

## Conclusion

Liberia has made remarkable progress in restoration of immunization service delivery following significant decline due to the devastating EVD outbreak in 2014-2015. There was improvement in immunization coverage for all antigens and reduced dropout rates at the national level. Key drivers for this can be associated with implementation of the immunization program within the GVAP framework and guidelines. However, there are still gaps within the system that need to be addressed in order to ensure maximum benefits from the immunization program. Some of these gaps include sub-optimal performance of immunization coverage and high drop-out rates at the county levels. Additionally, the quality of data for immunization service delivery is not optimal as there are discrepancies between administrative data and WUENIC data. The MCV coverage is still below elimination targets with sub-optimal population immunity and yet the country only has one dose of MCV in the immunization program. Nevertheless, Liberia has demonstrated strong capacity to adopt new technologies and vaccines into the immunization program with capacity to improve the health system and quality of service delivery. The primary strategies for successful introduction were country ownership, adaptation to country context and implementation within existing health system structures.

### What is known about this topic

- Impact of the Ebola outbreak on routine immunization with decline in vaccination coverage and immunity levels in the 3 most affected countries (Liberia, Sierra Leone and Guinea);
- There were recurrent outbreaks of VPDs and increases vulnerability towards importation of wild polio virus;
- There was emphasis on rebuilding strong health systems in the affected countries to redress the impact of the outbreak and strengthen health system capacity to better handle similar adverse events in the future.

### What this study adds

- Experience of Liberia on strengthening routine immunization following EVD outbreak control;
- Strategies and introduction of new technologies used to improve routine immunization in Liberia post Ebola outbreak;
- Existing gaps that needs improvement in Liberian context.

## Competing interests

The authors declare no competing interest.
